# A Hydrophobic Derivative of Ciprofloxacin as a New Photoinitiator of Two-Photon Polymerization: Synthesis and Usage for the Formation of Biocompatible Polylactide-Based 3D Scaffolds

**DOI:** 10.3390/polym13193385

**Published:** 2021-10-01

**Authors:** Kseniia N. Bardakova, Yaroslav V. Faletrov, Evgenii O. Epifanov, Nikita V. Minaev, Vladislav S. Kaplin, Yuliya A. Piskun, Polina I. Koteneva, Vladimir M. Shkumatov, Nadezhda A. Aksenova, Anastasia I. Shpichka, Anna B. Solovieva, Sergei V. Kostjuk, Peter S. Timashev

**Affiliations:** 1 Institute of Photonic Technologies, Research Center “Crystallography and Photonics”, Russian Academy of Sciences, 2 Pionerskaya St., Troitsk, 108840 Moscow, Russia; rammic0192@gmail.com (E.O.E.); minaevn@gmail.com (N.V.M.); timashev.peter@gmail.com (P.S.T.); 2 World-Class Research Center “Digital Biodesign and Personalized Healthcare”, Sechenov University, 8-2 Trubetskaya St., 119991 Moscow, Russia; shpichka_a_i@staff.sechenov.ru; 3 Research Institute for Physical Chemical Problems of the Belarusian State University, Minsk, Belarus 14 Leningradskaya St., 220030 Minsk, Belarus; yaroslav82@tut.by (Y.V.F.); piskunyu@gmail.com (Y.A.P.); biopharm@bsu.by (V.M.S.); kostjuks@bsu.by (S.V.K.); 4 Department of Chemistry, Belarusian State University, 14 Leningradskaya St., 220006 Minsk, Belarus; 5 Semenov Federal Research Center of Chemical Physics, Russian Academy of Sciences, 4 Kosygina St., 119991 Moscow, Russia; vladislav.s.kaplin@gmail.com (V.S.K.); naksenova@mail.ru (N.A.A.); ann.solovieva@gmail.com (A.B.S.); 6 Institute for Regenerative Medicine, Sechenov University, 8-2 Trubetskaya St., 119991 Moscow, Russia; koteneva.polina@yandex.ru; 7 Department of Chemistry, Lomonosov Moscow State University, 1-3 Leninskiye Gory, 119991 Moscow, Russia

**Keywords:** two-photon polymerization, scaffold, photoinitiator, ciprofloxacin, photopolymerization, star-shaped polylactide, fluorescence quantum yield, post-curing, mechanical properties

## Abstract

A hydrophobic derivative of ciprofloxacin, hexanoylated ciprofloxacin (CPF-hex), has been used as a photoinitiator (PI) for two-photon polymerization (2PP) for the first time. We present, here, the synthesis of CPF-hex and its application for 2PP of methacrylate-terminated star-shaped poly (D,L-lactide), as well a systematic study on the optical, physicochemical and mechanical properties of the photocurable resin and prepared three-dimensional scaffolds. CPF-hex exhibited good solubility in the photocurable resin, high absorption at the two-photon wavelength and a low fluorescence quantum yield = 0.079. Structuring tests showed a relatively broad processing window and revealed the efficiency of CPF-hex as a 2PP PI. The prepared three-dimensional scaffolds showed good thermal stability; thermal decomposition was observed only at 314 °C. In addition, they demonstrated an increase in Young’s modulus after the UV post-curing (from 336 ± 79 MPa to 564 ± 183 MPa, which is close to those of a cancellous (trabecular) bone). Moreover, using CPF-hex as a 2PP PI did not compromise the scaffolds’ low cytotoxicity, thus they are suitable for potential application in bone tissue regeneration.

## 1. Introduction

The two-photon polymerization (2PP) technology allows real 3D printing at a sub-100-nm resolution [1,2,3,4]. Due to the use of high-intensity femtosecond laser pulses, the two-photon absorption and subsequent polymerization occur in an extremely localized focal volume. In comparison with another widely known rapid prototyping technique—stereolithography, in which photopolymerization is confined to the surface of the photosensitive material—2PP offers certain advantages. Most importantly, in the 2PP process, the reduction of unwanted thermal reactions provides a spatiotemporal control over the photochemical initiation reactions [5] that makes 2PP attractive for a vast range of applications, including photonics, microfluidics, biomedical applications, or sensing.

There are two crucial components in a photocurable resin for 2PP: a photoinitiator (PI) and a monomer (or an oligomer). At the early stages of the 2PP method development, one-photon PIs, such as Irgacure 369, Irgacure 184, etc., were usually used due to their commercial accessibility [6,7]. These conventional one-photon PIs usually have high radical generation efficiency, but their two-photon absorption cross-section is quite low, therefore a high excitation power and a long exposure time were required, which often resulted in damage to the polymeric structures [8,9]. Although many PIs have been developed, over the last decade, to increase two-photon absorption cross-sections, the key factors for 2PP’s initiation efficiency are often neglected. The effective 2PP PIs should have low fluorescence quantum yields, as well as good photostability and solubility in the photocurable resin. In addition, they should provide a high quantum yield in the generation of active moieties, absorb at the two-photon wavelength and be transparent at the laser wavelength [8,10,11,12,13,14].

Compounds with a low fluorescence quantum yield are preferred for 2PP, as this leads to higher population of the active triplet state for initiating the polymerization. It has been shown, recently, that the presence of a carbonyl group in PI results in the reduction of the fluorescence quantum yield due to its strong electron-withdrawing effect, as well as facilitation of the intersystem crossing, owing to the efficient spin-orbital coupling [15,16]. Following this idea, a series of biocompatible π-expanded ketocoumarins were synthesized, showing high efficiency as initiators for 2PP [17]. In the present work, we studied a derivative of an antibiotic ciprofloxacin as a new 2PP PI. Similarly to ketocoumarins, ciprofloxacin has π-expanded conjugation, involving a carbonyl group and also demonstrates the ability to generate free radicals [18,19,20]. Methacrylate-terminated star-shape poly (D,L-lactide) [21] was used as a basic photosensitive material. The main drawback of ciprofloxacin is its low lipophilicity, which excludes its use as a PI for polylactide scaffold fabrication by the 2PP technique. Therefore, a hydrophobic derivative of ciprofloxacin, hexanoylated ciprofloxacin (CPF-hex), was obtained using hexanoic anhydride [22]. Such a modification increases the lipophilicity of initial ciprofloxacin. The synthesized ciprofloxacin derivative was compared to the commercial photoinitiator 4,4′-bis (diethylamino) benzophenone (Bis-b). We have previously shown that three-dimensional scaffolds, based on a methacrylate-terminated star-shape polylactide and Bis-b, are biocompatible and induce the differentiation of mesenchymal stem cells toward the osteogenic lineage [21,23].

Furthermore, one of this study’s objectives was to assess the effect of UV post-processing on the mechanical properties of three-dimensional (3D) scaffolds based on the investigated compositions. The photocurable resins for 2PP are usually viscous, and polymerization occurs within a small volume of a material called a voxel. Therefore, in a scaffold there are unreacted double bonds trapped between voxels, and the distribution of the components is inhomogeneous [24]. That is, in addition to the anisotropic shrinkage of materials and discrepancy between the computer model and the formed scaffold, which often accompany the 2PP method [25], the unpolymerized monomers can cause cytotoxic effects and reduce the elastic modulus of the sample. To eliminate or at least mitigate these complications, several approaches have been proposed, such as using different shrinkage guides, increasing the laser exposure time, adjusting the laser power, applying different post-processing methods, etc. The most wide-spread approach for the post-processing of 3D printed scaffolds is UV curing, which is also effective for open-cell submicron structures [26,27].

**Figure 1:**
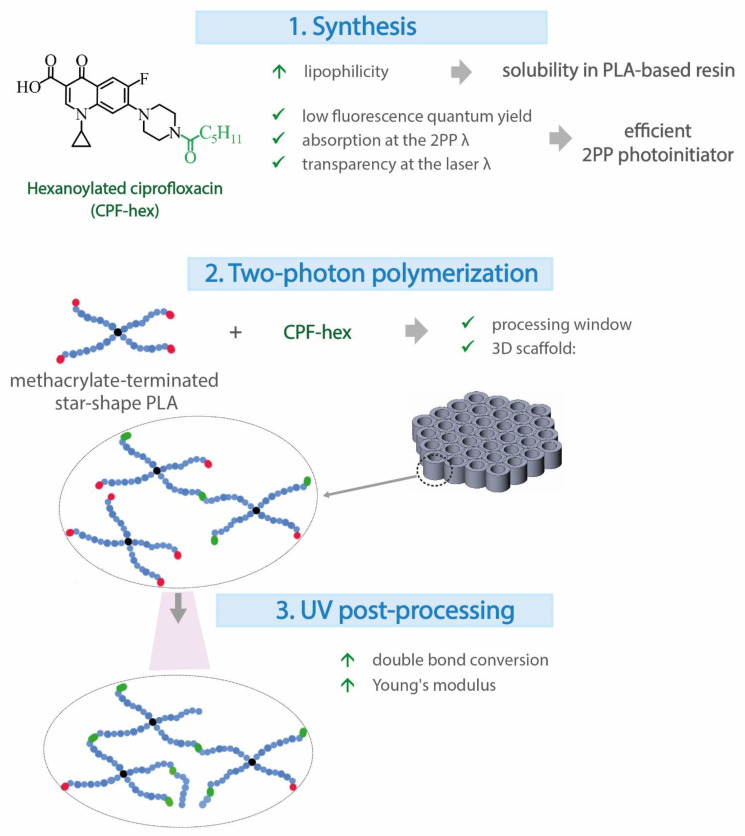
Basic stages of the study.

Based on the above, this study is divided into three parts (Figure 1). First, we performed the introduction of a hydrophobic group into ciprofloxacin to obtain an efficient 2PP PI with a low fluorescence quantum yield and excellent solubility in the star-shaped polylactide-based resin. The 2PP processing window, i.e., the laser power and the scan speed, which results in stable, non-damaged structures, was defined, and 3D scaffolds were fabricated. Then, we studied the thermal stability, as well as the physicochemical and mechanical properties of the scaffolds including those after the UV post-processing.

## 2. Materials and Methods

### 2.1. Synthesis of CPF-hex

Ciprofloxacin hydrochloride donated by Zhejiang LangHua pharmaceutical Co., Ltd. (Linhai, China) (100 mg, 272.5 µmol, 1 eq.) was mixed with 1 mL of dimethyl sulfoxide (Sigma, St. Louis, MO, USA, 99.9%), 0.5 mL of pyridine (Sigma, St. Louis, MO, USA, 99.8%) and hexanoic anhydride (100 µL, 433 µmol, 1.6 eq., Sigma, St. Louis, MO, USA, 97%) at room temperature followed by heating up to 50–60 °C during 5 min. Then, the mixture was allowed to cool down to room temperature, and 10 mL of benzene (Ecos-1, Moscow, Russia, >99%) was added dropwise, resulting in the crude product precipitation. The precipitate was dissolved in acetone (20 mL, Ecos-1, Moscow, Russia, 97%) using sonication (5 min, Elmasonic S30H, Elma Ultrasonic Technology, Singen, Germany) and then placed to a SiO_2_ column for chromatographic purification. Appropriate fractions were combined and dried using a rotary evaporator that resulted in 90 mg (69% yield) of a slightly yellowish solid.

CPF-hex (1-cyclopropyl-6-fluoro-7-(4-hexanoylpiperazin-1-yl)-4-oxo-1,4-dihydroquinoline-3-carboxylic acid), ^1^H NMR (400 MHz, CDCl_3_) δ (ppm): 8.7 (s, 1H), 7.97–7.95 (d, 1H), 7.35–7.34 (d, 1H), 3.84–3.70 (dm, 4H), 3.54 (m, 1H), 3.34–3.25 (dm, 4H), 2.36 (t, 2H), 1.64 (m, 2H), 1.38–1.30 (m, 6H), 1.2 (m, 2H), 0.88 (m, 3H) (Figure S1). ^19^F NMR (400 MHz, CDCl_3_) δ (ppm): −(121,03–121,07) (dd, 1F) (Figure S2); ESI-MS m/z: found 430.25 for ions [M + H]^+^, calculated 430.21 for C_23_H_29_FN_3_O^4+^ (notably, also found 431.25 at 25% level reflecting the correct isotopes composition) (Figure S3); TLC (SiO_2_, acetone: acetic acid [5 : 1 by volume]) Rf: 0.95 vs. 0.10 for ciprofloxacin.

### 2.2. Preparation of Photocurable Resins

The basic component of our photocurable resins was tetrafunctional branched poly (D,L-lactide) (Mn(GPC) = 3528 g/mol, Mw/Mn = 1.29) synthesized according to the methods described in our previous study [21]. The photocurable resins were prepared by the following technique. CPF-hex (or Bis-b) was dissolved in tetrahydrofuran (THF, Sigma, St. Louis, MO, USA, ≥99.9%) to reach a 0.7 wt% photoinitiator solution, then the tetrafunctional methacrylate functionalized polylactide (25 wt% in the final composition) was added. The photocurable resins were left under stirring overnight. After that, the polylactide-based photocurable resin (about 100 µL) was transferred to a spacer and used to produce scaffolds via 2PP.

### 2.3. Scaffolds Fabrication via 2PP

**Figure 2:**
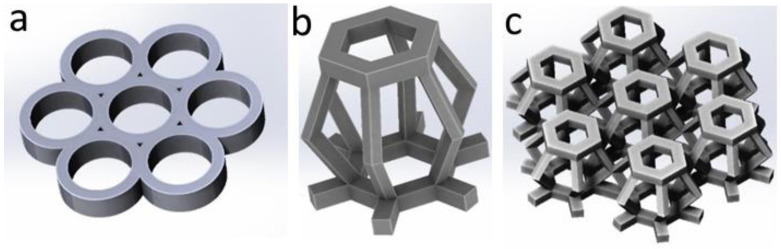
(**a**) 3D model for the structuring tests; (**b**) a structural element of (**c**) a hexagonal beam structure.

The scaffolds were produced using a microfabrication femtosecond laser system (Laser nanoFab GmbH, Hannover, Germany), which delivers 200-fs pulses at a 70 MHz repetition rate. In order to assess the optimal processing window of the resins, spatially defined structures (Figure 2a; a set of cylinders with an inner diameter of 180 μm, an outer diameter of 250 μm, a height of 80 μm, 1 μm hatch distance, 2 μm layer distance) were fabricated. The writing speed was 25,000 µm/s. The laser power was varied in a range of 10–80 mW. To wash a scaffold from the uncrosslinked resin, it was placed into dichloromethane or THF for 1 h. Using a hexagonal beam structure as a structural element (Figure 2b), 3D scaffolds with complex geometry were fabricated as a result (Figure 2c).

### 2.4. Analytical Methods

#### 2.4.1. Spectroscopic Methods

UV-Vis absorption spectra were recorded with a Cary-50 spectrophotometer (Varian Inc., Palo Alto, CA, USA) at a concentration of 800 μg/mL.

Fluorescence spectra were recorded with a Fluoromax Plus fluorescence spectrometer (Horiba Jobin Yvon, Edison, NJ, USA). The relative fluorescence quantum yields were measured using anthracene (quantum yield = 0.27 in ethanol) as a reference standard [28].

IR spectra were acquired with a FT-IR Spectrum Two spectrometer (PerkinElmer Inc., Waltham, MA, USA) in the ATR mode with a resolution of 4 cm^−1^ and averaged over 8 scans. The initial star-shaped methacrylate functionalized polylactide and the scaffolds after 2PP were analyzed using a GladiATR single-reflection accessory (Pike Technologies, Madison, WI, USA) equipped with a monolithic diamond crystal (incident angle = 45°, refractive index = 2.4). Each spectrum was calibrated by the intensity of the C–O stretching vibration (1084 cm^−1^) and underwent the Fourier-transform smoothing procedure by four points.

#### 2.4.2. Optical Microscopy SEM

To estimate the optimal power of the 2PP method and the completeness of washing from the uncured photosensitive composition, a KOZO XJF900 (Kozo Optics, Nanjing, China) microscope and SEM with a Phenom Pro X. (Phenom-World, Eindhoven, the Netherlands) instrument were used.

#### 2.4.3. DSC

Differential scanning calorimetry (DSC) measurements were performed using an STA 6000 simultaneous thermal analyzer (PerkinElmer Inc., Waltham, MA, USA). Samples for DSC experiments (about 10 mg) were encapsulated in standard PerkinElmer pans and heated in a nitrogen medium at a gas flow rate of 20 mL/min and a linear heating rate of 10 °C/min.

#### 2.4.4. Nanoindentation

To study the mechanical characteristics, the square scaffolds of side width 3 mm and height 400 μm were formed. The scaffolds were additionally irradiated for 10 min with a UV light-emitting diode (Epileds, Tainan, Taiwan) with a wavelength of 365 nm at the intensity of 3.9 mW/cm^2^. The local mechanical characteristics for the initial scaffolds and for the irradiated scaffolds were measured with a Piuma Nanoindenter (Optics11, Amsterdam, the Netherlands). A cantilever with the spring constant of 126.1 N/m and the tip radius of 56 µm was used. The measurements were performed in distilled water at 37 °C, the scan size was 1 × 1 mm^2^ with a 100 µm step by the X and Y axes. Using the Hertzian model, we calculated the effective Young’s modulus and created distribution maps of the Young’s moduli over the surface.

### 2.5. Cytotoxicity

The material cytotoxicity was assessed using Live/Dead (Sigma Aldrich, Steinheim am Albuch, Germany) staining and AlamarBlue (Invitrogen, Carlsbad, CA, USA) and PicoGreen (Invitrogen, Carlsbad, CA, USA) assays. We used mesenchymal stromal cells (MSC) derived from the gingiva. The cells were cultured as described elsewhere [29] and monitored using a phase contrast microscope. A scaffold was inoculated with 50,000 cells and stained with calcein-AM (green, live cells) and propidium iodide (red, dead cells) in 5 days. Then it was analyzed using an LSM 880 laser scanning confocal microscope equipped with an AiryScan module and GaAsP detector (Carl Zeiss, Jena, Germany). For colorimetric assays, the material extract was prepared as previously described [30] and used to prepare serial dilutions. The sample solution or sodium dodecyl sulfate (SDS, positive control) were added to the cells cultured in a 96-well plate for 24 h at a temperature of 37 °C.

## 3. Results and Discussion

### 3.1. Synthesis of CPF-hex Optical Properties 2PP Fabrication

**Figure 3:**
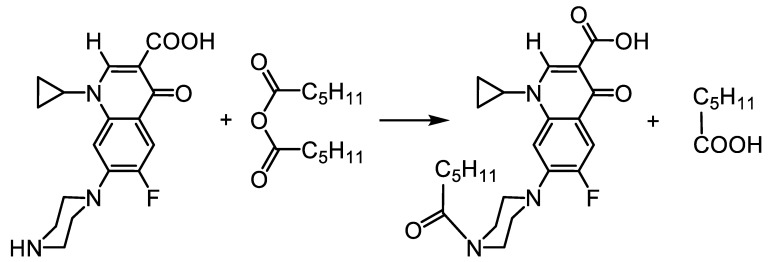
Synthetic strategy for the preparation of CPF-hex.

The N-hexanoylated ciprofloxacin derivative (CPF-hex) was synthesized by the reaction of ciprofloxacin with 1.5 equivalents of hexanoic anhydride (Figure 3). The structure of the synthesized compound was confirmed by ^1^H, ^19^F NMR and mass spectrometry (ESI-MS) (see Section 2.1 and Figures S1–S3).

**Figure 4:**
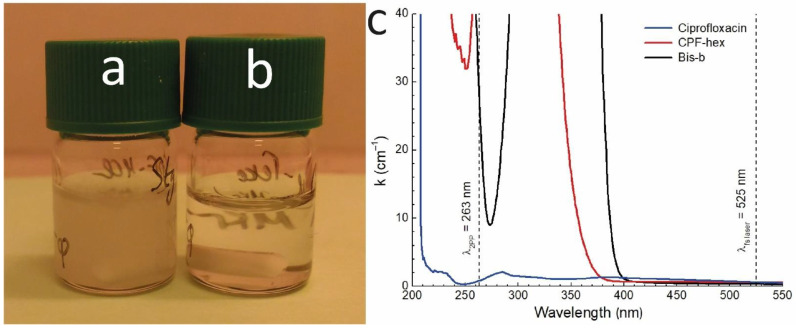
The ciprofloxacin (**a**) and CPF-hex (**b**) solubility in THF (800 µg/mL). (**c**): Absorption spectra of ciprofloxacin, hexanoylated ciprofloxacin (CPF-hex), 4,4′-bis (diethylamino) benzophenone (Bis-b) in THF.

The introduction of a hydrophobic group into ciprofloxacin increased its lipophilicity. The calculated lipophilicity values of ciprofloxacin and its hexanoylated derivative were 1.78 and 3.26, respectively. As compared with unmodified ciprofloxacin, CPF-hex showed greater solubility in dichloromethane and THF – suitable solvents for polylactide (Figure 4a,b). At the same time, the CPF-hex solutions were transparent at the femtosecond laser wavelength (λ = 525 nm) and had an intense absorption at the 2PP wavelength (λ = 263 nm) (Figure 4c).

Note that the linear absorption coefficient of CPF-hex at the 2PP wavelength was higher than that of Bis-b. Many studies on the synthesis of 2PP PIs focus on the development of compounds with high nonlinear absorption coefficients or large two-photon absorption cross-sections. To measure them, the Z-scan technique is used. However, the efficiency of 2PP is complex and multifactorial, with several processes taking place over different time scales. For example, a high fluorescence yield can lead low 2PP efficiency, since relaxation of the excited state via fluorescence and transition to the triplet state are competing pathways [10]. To reduce the fluorescence contribution, systems with low-lying *n*-π* states, e.g., with acceptor carbonyl groups in their structure, were synthesized. Similar to many photoinitiators of radical polymerization, ciprofloxacin has a carbonyl group, which is directly bound and conjugated to the aromatic system. According to the simplified mechanism of photoactivation of such compounds, it may generate a biradical upon absorption of light, due to the homolytic cleavage of one of the π–π bonds. Further intramolecular processes and intermolecular interactions lead to the destruction of the piperazine cycle, defluorination and decarboxylation. The transition of fluoroquinolones to the excited state is generally described as a transition from the S_0_ state to the excited S_1_ state, then, to the triplet T_1_ state by intersystem crossing. The radiative decay from the S_1_ state to S_0_ is responsible for the fluorescence (with the emission maximum at 450 nm), while the excitation energy transfer to oxygen molecules is provided by the T_1_ state [31]. The generation of radicals from the excited state and their mobility also affect the efficiency of 2PP.

In practical terms, all the factors are summed up in the processing or fabrication window, which is related to the intensity of the laser radiation. The processing window is defined by two thresholds [32,33]. The first one is the polymerization threshold, below which polymerization is not observed. It is determined by the physicochemical properties and concentration of PI. Further increasing the light intensity may lead to the reaching of the damage threshold, above which the material boils, forming gas bubbles. Photocurable compositions with a larger processing window allow the use of different voxel sizes, which may be interesting in regard to the formation of 3D scaffolds with specifically distributed surface and mechanical properties.

**Figure 5:**
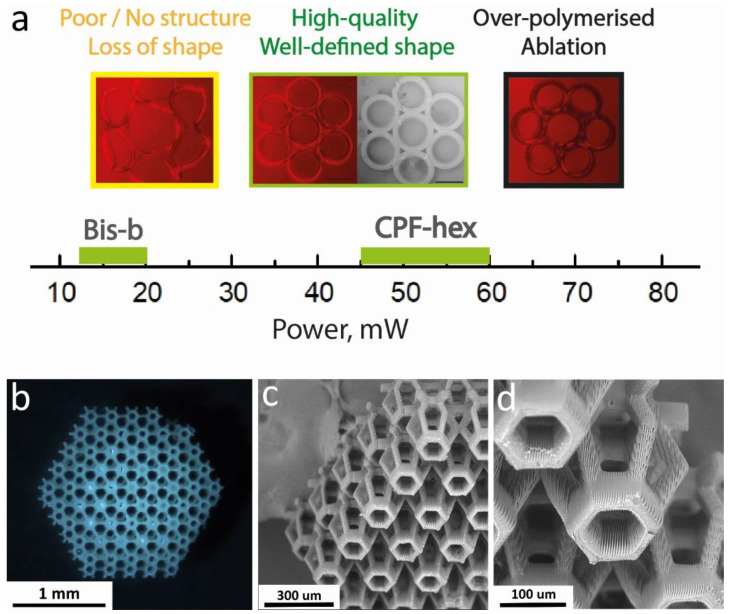
(**a**): The 2PP structuring test results of Bis-b and CPF-hex. The power range required to create high-quality structures is shown; (**b**–**d**): a 3D scaffold fabricated by 2PP. CPF-hex was used as a photoinitiator.

Several approaches are used to evaluate the processing window and the photoinitiation efficiency of 2PP PIs, such as single-line writing or the fabrication of individual test structures. In this study, the efficiency of CPF-hex as a 2PP PI was evaluated by the fabrication of a three-dimensional structure consisting of seven cylinders (Figure 2a). The threshold values of the average laser power were determined using a constant writing speed of 25,000 µm/s. As shown by the structuring tests, CPF-hex had a high-quality processing window of 45–60 mW. At a power lower than 45 mW, photopolymerization is characterized by low efficiency, proven by the creation of ill-defined structures with poor mechanical properties. One may note the high heterogeneity and significant ablation of the fabricated structures at a power higher than 60 mW (Figure 5a). In additional experiments, polylactide scaffolds in the presence of CPF-hex as a photoinitiator were fabricated at an average laser power of 50 mW. The obtained scaffolds were stable after washing away the uncured resin; the mechanical properties allowed their manipulation with tweezers. The selected parameters of 2PP also allowed creating 3D scaffolds with a complex geometry, using a truncated ellipsoid as a structural element (Figure 5b). The SEM-micrographs (Figure 5c,d) demonstrate good reproducibility of the preset computer model (Figure 2c) and a pronounced three-dimensional relief that may promote adhesion of cells cultured at the scaffold’s surface.

A photocurable resin, based on methacrylate-terminated star-shaped polylactide and Bis-b as a photoinitiator, was used as a reference standard due to good results we had obtained previously [21]. In this case, the processing window was narrower (12–20 mW; Figure 5a), however, a low polymerization threshold was observed.

**Figure 6:**
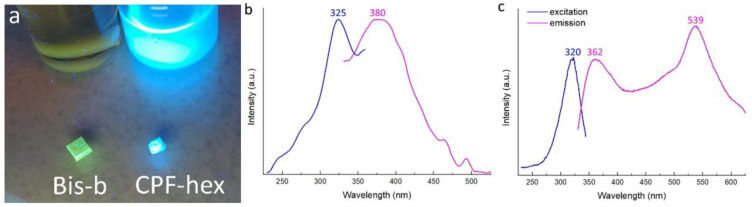
(**a**): The photocurable resins and the scaffolds under UV-LED 365 nm. The fluorescence spectra of the scaffolds with CPF-hex (**b**) and Bis-b (**c**).

The fluorescence spectra for scaffolds with CPF-hex and Bis-b were recorded (Figure 6) and their fluorescence quantum yields were determined.

Ciprofloxacin, being a representative quinolone carboxylic acid derivative, generally shows a complex fluorescence behavior depending on the solvent polarity and pH. It has native fluorescence emission at 440 nm (quantum yield = 0.28) upon excitation at 278 nm in water [34].

The excitation and emission maxima of scaffolds with CPF-hex were 325 and 380 nm, respectively (Figure 6b). The retention of the ability to fluoresce for scaffolds with CPF-hex may be related to two reasons. First, the unreacted CPF-hex is not completely removed after the washing procedure. It is also possible that the photoinduced formation of CPF-hex radicals, which have reacted with methacrylate groups, is not accompanied by the destruction of the fluoroquinolone core.

Scaffolds with Bis-b (Figure 6c) had the emission maxima at 362 and 539 nm upon excitation at 320 nm. Note, that the scaffold with CPF-hex had about a 115-fold lower fluorescence intensity in comparison with the scaffold with Bis-b. However, the opposite situation was observed in the case of chloroform solutions: the CPF-hex solution fluoresced more intensely than did the solution with Bis-b. In the solid state, ciprofloxacin generally suffers from aggregation-caused quenching [35]. Bis-b is a specific luminogen with a donor-acceptor structure: the acceptor carbonyl group is connected to two donor diethylphenylamine units. In contrast to CPF-hex, Bis-b exhibits the aggregation-induced emission enhancement effect in its solid state. This phenomenon is presented in detail in [36]. Autofluorescence of polymer scaffolds allows studying their further biodegradation in vivo without the need of developing complicated histological procedures and sacrificing experimental animals [21]. Nevertheless, for biomedical applications, the development of a non- or low-fluorescent scaffold that does not impede or hinder the imaging of the created tissues and organs is a necessity. To address the drawback of autofluorescence, several approaches are proposed [37]: photobleaching of polymers, the use of infrared fluorophores or molecular quenchers, the use of photoinitiator-free resins for 2PP, etc. Note also that bone tissues emit characteristic green fluorescence upon excitation by a UV-blue light [38]. Thus, such scaffolds with CPF-hex may be used in the future for bone regeneration due to the possibility of distinction between the scaffold and the surrounding tissue.

Low fluorescence quantum yields are desirable for 2PP PIs, since fluorescence is a common loss channel, competing with the initiation of polymerization. The fluorescence quantum yields of CPF-hex and Bis-b (used as the golden standard for several published structures) in chloroform were determined to be 0.079 and 0.003, respectively. Similar values of the fluorescence quantum yield of 2PP PIs have been obtained in other studies. For example, the authors of [39] synthesized new thioxanthone-based photoinitiators, characterized by a low fluorescence quantum yield up to 0.081. The authors of [11] showed the photoinitiation efficiency of C_2v_ symmetrical anthraquinone derivatives with the fluorescence quantum yield up to 0.043. In [14], other similar C_2v_-symmetrical 2PP PIs were studied (the emission quantum yields = 0.2). The structuring tests revealed the efficiency of these materials as 2PP PIs.

It can be concluded that, although fluorescence-based techniques are prone to larger errors, when the fluorescence quantum yield is very small (as e.g., for Bis-b), the results show that the ciprofloxacin derivative exhibits a low fluorescence quantum yield. This finding, as well as the intense self-absorption and relatively broad processing window, might make CPF-hex a promising candidate as a photoinitiator for 2PP.

### 3.2. IR Spectra and Differential Scanning Calorimetry

**Figure 7:**
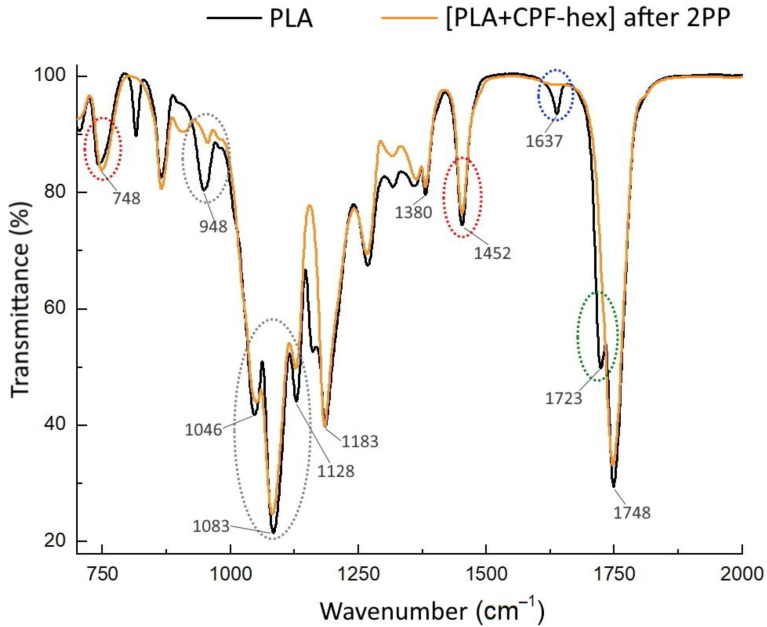
The IR spectra of the initial methacrylate-terminated star-shaped polylactide (PLA) and the polylactide-based resin after the 2PP processing ([PLA + CPF-hex] after 2PP).

The IR spectra of the initial methacrylate-terminated star-shaped polylactide and the 2PP scaffold with CPF-hex (Figure 7) contain absorption bands typical for lactide chains, namely: stretching vibration of the carbonyl group C = O (1748 cm^−1^), bending vibrations of CH_3_ (1452 and 1380 cm^−1^), stretching vibrations of C–O–C (bands in the range from 1046 to 1183 cm^−1^), etc. [2,23]. At the same time, the IR spectra may be used to observe the changes that have occurred with the methacrylate-terminated star-shaped polylactide after photo-crosslinking. The three-dimensional network formation occurs primarily through the reaction of the terminal methacrylate groups in the polylactide, therefore, one can see changes in the bands of their spectra, as well as in the bands of the conjugated functional groups. Thus, a clearly visible sign of photo-crosslinking is the disappearance of the peak at 1637 cm^−1^, assigned to the stretching vibrations of the terminal methacrylate groups (blue section in Figure 7). Furthermore, we also relate the absence of a peak at about 1723 cm^−1^ (stretching vibration of carbonyl groups in unsaturated esters) to the loss of C = C groups (green section in Figure 7). Note, also, that the peak at 1452 cm^−1^ (deformation vibrations of CH_2_-, CH_3_-groups) for the scaffold is less intense than that for the initial methacrylate-terminated star-shaped polylactide and the position of the rocking vibrations of CH_2_ increases from 741 to 748 cm^−1^, which may be attributed to the changes in the polymer network (red sections in Figure 7). In addition, there is attenuation in the intensity of peaks of symmetrical and asymmetrical stretching of C–O–C (948, 1046, 1083, 1128 cm^−1^) (gray sections in Figure 7).

**Figure 8:**
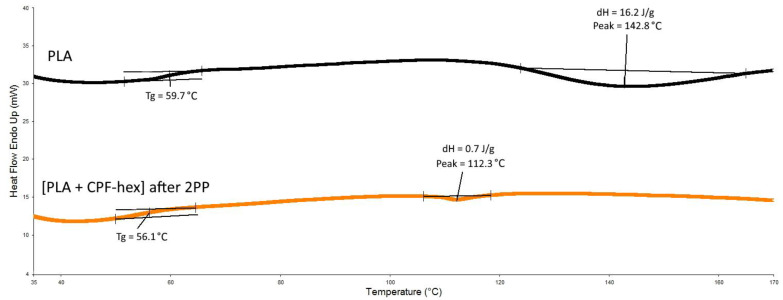
DSC curves of the initial methacrylate-terminated star-shaped polylactide (PLA) and the polylactide-based resin after the 2PP processing ([PLA + CPF-hex] after 2PP).

The thermal properties of the polylactide before and after the 2PP structuring were evaluated using DSC and TG methods. Methacrylate-terminated star-shaped polylactide is not stereoregular, it is a completely amorphous polymer, therefore the samples before and after 2PP demonstrated second-order transitions, such as glass transition temperatures (T_g_) equal to 59.7 and 56.1 °C, respectively (Figure 8; Table 1). It was expected that after photo-crosslinking, due to the formation of local cross-links (knots), a material with a higher T_g_ than that of the initial star-shaped polylactide would be obtained. The formation of covalent bonds limits the thermal motion of adjacent links, the cross-linked system becomes more rigid, therefore, the transition to the rubbery state should occur at higher temperatures.

In our experiment, the change in the polarity of the polymer system most probably is a decisive factor in terms of the influence on T_g_, rather than a decrease in the chain flexibility. This means that the screening effects for the polar bonds (C–O–C, O=C–O) in the photocured system are observed, therefore, T_g_ decreases.

Note also that, in the first scan (Figure 8), exothermic peaks are observed: for the initial methacrylate-terminated star-shaped polylactide—at 142.8 °C (dH = 16.2 J/g), for the scaffold after 2PP—at 112.3 °C (dH = 0.7 J/g). These peaks may be related to thermal cross-linking of unreacted methacrylate groups, since both the one-photon and 2PP methods do not lead to the complete conversion of photosensitive double bonds. For example, one study [33] reports the typical achievable values of the degree of conversion as ranging from 60 to 80%. The incomplete double bond conversion is shown by the lower dH for the cross-linked sample, as well as the absence of peaks in this region after the second scan (Figure S4). Among the possible causes for the T_g_ decrease after the cross-linking, the presence of the unreacted composition in the sample, which plays a role of a distinctive plasticizer, may also be mentioned.

The obtained thermogravimetry results are presented in Table 1. It has been shown that the thermal degradation occurs in the same temperature range for both the initial and photo-crosslinked polylactide. Moreover, the obtained values for the degradation temperatures are close to those obtained for linear polylactides [40,41]. This indicates that the introduction of methacrylic groups or the three-dimensional network formation do not significantly influence the thermal degradation of polylactide.

### 3.3. Mechanical Properties

Appropriate mechanical properties are one of the key requirements of three-dimensional scaffolds for their subsequent in vitro and in vivo applications. Scaffolds need to maintain the structural integrity after implantation in the host tissue until regeneration. The scaffold’s mechanical properties will affect the biomaterial’s degradability, cell behavior and signaling pathways [42,43].

**Figure 9:**
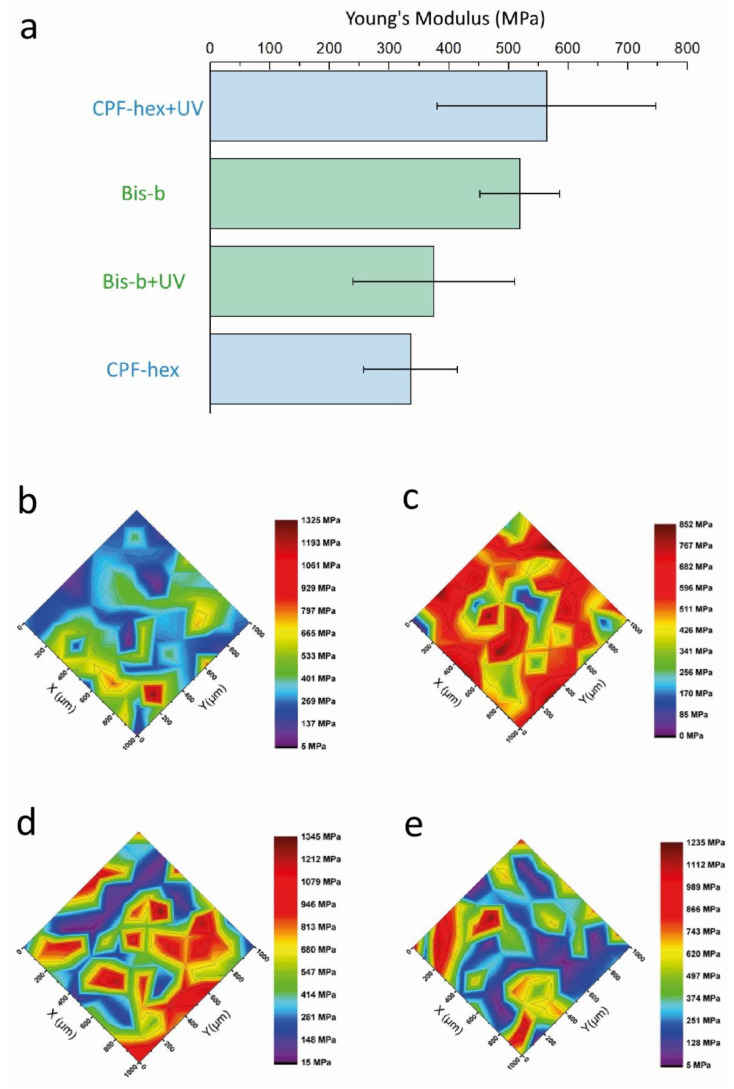
(**a**): Young’s modulus measured by nanoindentation (data are represented as the mean ± SD). The Young’s moduli distribution over the surface of the initial scaffolds with CPF-hex (**b**), Bis-b (**c**) and UV post-cured scaffolds with CPF-hex (**d**), Bis-b (**e**).

In the case of 2PP-fabricated scaffolds, their mechanical properties are significantly determined by the composition of the photocurable resin and the laser fabrication parameters. Both factors affect the degree of double bond conversion and, hence, the elastic modulus of the scaffold. In our work, we studied the effect of UV irradiation on the local elastic modulus of scaffolds. It was shown that the more pronounced increase in mechanical properties after the UV post-curing was observed for scaffolds based on methacrylate-terminated star-shaped polylactide and CPF-hex: the Young’s modulus increased by 40%, from 336 ± 79 MPa to 564 ± 183 MPa. For scaffolds with Bis-b, in contrast, the UV post-treatment did not lead to a significant growth of the elastic modulus, while the spread of values increased (Figure 9). Note, that UV post-processing may result in two opposite effects. In addition to the cross-linking of the uncured photosensitive composition and removal of the solvent that leads to higher values of elastic moduli, the high-energy UV light may also break bonds and degrade polymers [44]. Thus, an increase in the spread of values may be attributed to these two competing effects.

We have previously shown [21] that three-dimensional scaffolds based on methacrylate-terminated star-shape polylactide and Bis-b induce the differentiation of mesenchymal stem cells toward the osteogenic lineage. In this study, the obtained scaffold’s mechanical properties are also close to the properties of the cancellous (trabecular) bone (equal to 0.1–1.0 GPa) [45]. Therefore, such 3D scaffolds may be further considered for bone regeneration. Note also that this resin was used in [29] for the formation of microcapsules upon UV irradiation. As was shown by mechanical testing, the elastic modulus did not exceed 80 MPa, which demonstrates the importance of selecting the optimal fabrication method for the appropriate mechanical properties.

### 3.4. Cytotoxicity of Scaffolds with CPF-hex or Bis-b

**Figure 10:**
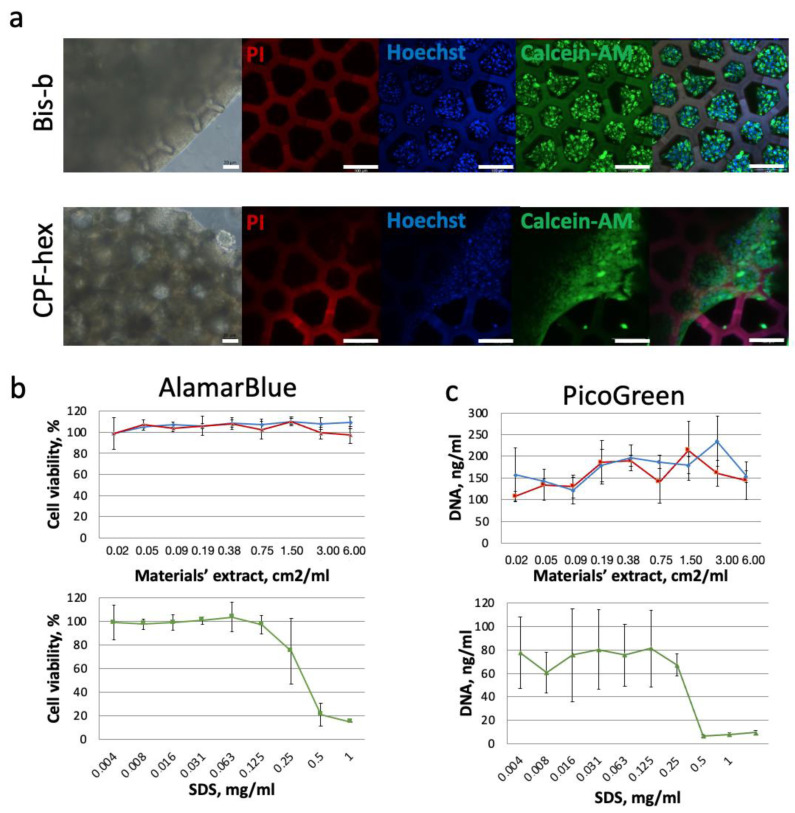
Cytotoxicity of the prepared scaffolds: (**a**) Phase contrast images (scale bar 20 µm) and live/dead staining of the MSC-seeded scaffolds (red—dead cells, blue—cell nuclei, and green—live cells; scale bar 100 µm); (**b**) AlamarBlue and (**c**) PicoGreen assays (blue—CPF-hex, red—Bis-b, and green—SDS (positive control).

After the seeding, we showed that MSC attached to a scaffolds’ surface and actively proliferated (Figure 10). The cells had typical morphology and formed multiple intercellular junctions. In 5 days, they spread into scaffolds’ pores and covered almost the whole top surface. While cultured on the scaffolds fabricated using CPF-hex, MSC formed the monolayer on its surface easily detaching during manipulations with this scaffold. This can be explained by the topography of the scaffold surface, which is relatively smooth (Figure 5). Most cells remained viable; only single dead cells were revealed (Figure 10). The AlamarBlue assay showed that the cell viability exceeded 70% for all the samples’ extracts that was confirmed by the PicoGreen assay.

## 4. Conclusions

In this study, we have developed a simple approach for the preparation of an efficient and non-toxic photoinitiator for 2PP, which consists in the introduction of a hydrophobic group into ciprofloxacin, a known commercial antibiotic. This simple modification allows achieving excellent solubility of this compound (CPF-hex) in the photocurable composition. In addition, the photophysical studies showed that CPF-hex exhibited high absorption at the two-photon wavelength, a low fluorescence quantum yield (0.079) and a relatively broad processing window. These properties make CPF-hex a promising candidate for application as a photoinitiator in 2PP printing. Finally, well-defined three-dimensional scaffolds were fabricated, characterized by good thermal stability, no cytotoxicity and a high Young’s modulus.

## Figures and Tables

**Table 1:** Thermal properties of the initial methacrylate-terminated star-shaped polylactide (PLA) and the polylactide-based resin after the 2PP processing ([PLA + CPF-hex] after 2PP).

Sample	T_g_, °C	T_10% mass__loss_, °C	Moisture Content at 100 °C, %
PLA	59.7	314	8
[PLA + CPF-hex] after 2PP	56.1	315	4
		

## Data Availability

The data presented in this study are available on request from the corresponding author.

## Supplementary Materials

The following are available online at https://www.mdpi.com/article/10.3390/polym13193385/s1, Figure S1: ^1^H-NMR spectra of CPF-hex; Figure S2: ^19^F-NMR spectra of CPF-hex; Figure S3: ESI-MS spectrum of CPF-hex; Figure S4: DSC curves of initial methacrylate-terminated star-shaped polylactide (PLA) and a polylactide-based resin after the 2PP processing ([PLA + CPF-hex] after 2PP) (the second scan).
