# Supplementary material for: A Hydrophobic Derivative of Ciprofloxacin as a New Photoinitiator of Two-Photon Polymerization: Synthesis and Usage for the Formation of Biocompatible Polylactide-Based 3D Scaffolds
Source: Polymers (Basel). 2021 Oct 1;13(19):3385. doi: 10.3390/polym13193385 (PMC8512357; doi:10.3390/polym13193385)
Supplement: Supplementary file 1 [file polymers-13-03385-s001.zip › polymers-1381561-supplementary.pdf]

Supplementary Material

A Hydrophobic Derivative of Ciprofloxacin as a New Photoinitiator of Two-Photon Polymerization: Synthesis and Usage for the Formation of Biocompatible Polylactide-Based 3D Scaffolds

Kseniia N. Bardakova [1, 2,*], Yaroslav V. Faletrov [3,4], Evgenii O. Epifanov [1], Nikita V. Minaev [1], Vladislav S. Kaplin [5], Yuliya A. Piskun [3,4], Polina I. Koteneva [6], Vladimir M. Shkumatov [3,4], Nadezhda A. Aksenova [5,6], Anastasia I. Shpichka [2,6,7], Anna B. Solovieva [5], Sergei V. Kostjuk [3,4,6] and Peter S. Timashev [1,5,6,7]

[1] Institute of Photonic Technologies, Research center "Crystallography and Photonics", Russian Academy of Sciences, 2 Pionerskaya st., Troitsk, 108840 Moscow, Russia; rammic0192@gmail.com (E.O.E); minaevn@gmail.com (N.V.M.); timashev.peter@gmail.com (P.S.T.)

[2] World-Class Research Center "Digital Biodesign and Personalized Healthcare", Sechenov University, 8-2 Trubetskaya st., 119991 Moscow, Russia; shpichka_a_i@staff.sechenov.ru (A.I.S.)

[3] Research Institute for Physical Chemical Problems of the Belarusian State University, Minsk, Belarus 14 Leningradskaya st., 220030 Minsk, Belarus; yaroslav82@tut.by (Y.V.F.); piskunyu@gmail.com (Y.A.P); biopharm@bsu.by (V.M.S.); kostjuks@bsu.by (S.V.K.)

[4] Department of Chemistry, Belarusian State University, 14 Leningradskaya st., 220006 Minsk, Belarus;

[5] Semenov Federal Research Center of Chemical Physics, Russian Academy of Sciences, 4 Kosygina st., 119991 Moscow, Russia; ann.solovieva@gmail.com (A.B.S.); naksenova@mail.ru (N.A.A.); vladislav.s.kaplin@gmail.com (V.S.K.)

[6] Institute for Regenerative Medicine, Sechenov University, 8-2 Trubetskaya St., 119991 Moscow, Russia;

[7] Department of Chemistry, Lomonosov Moscow State University, 1-3 Leninskiye Gory, 119991 Moscow, Russia

* Correspondence: arie5@yandex.ru;

Table of Contents:

Figure S1 ^{1}H-NMR spectra of CPF-hex.

Figure S2 ^{19}F-NMR spectra of CPF-hex.

Figure S3 ESI-MS spectrum of CPF-hex.

Figure S4 DSC curves of initial star-shaped polylactide (PLA) and a polylactide-based resin after the 2PP processing ([PLA+ CPF-hex] after 2PP) (the second scan).

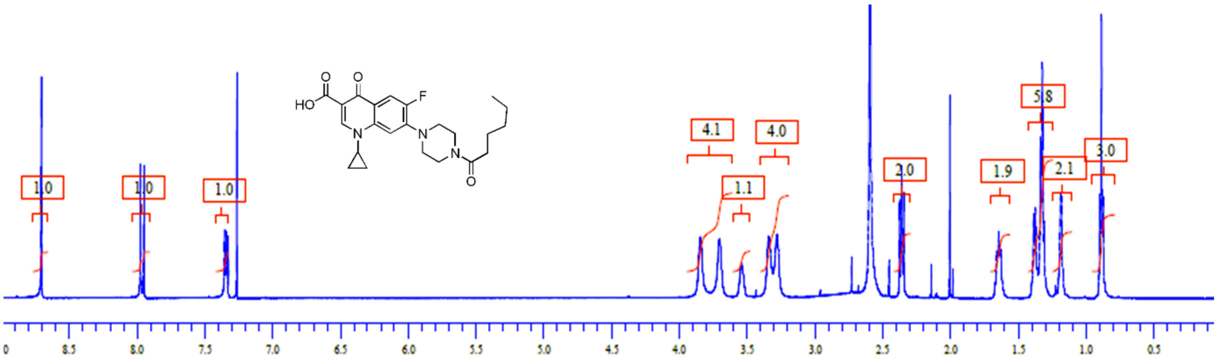

Figure S1. ^{1}H-NMR spectra of CPF-hex (1-cyclopropyl-6-fluoro-7-(4-hexanoylpiperazin-1-yl)-4-oxo-1,4-dihydroquinoline-3-carboxylic acid).

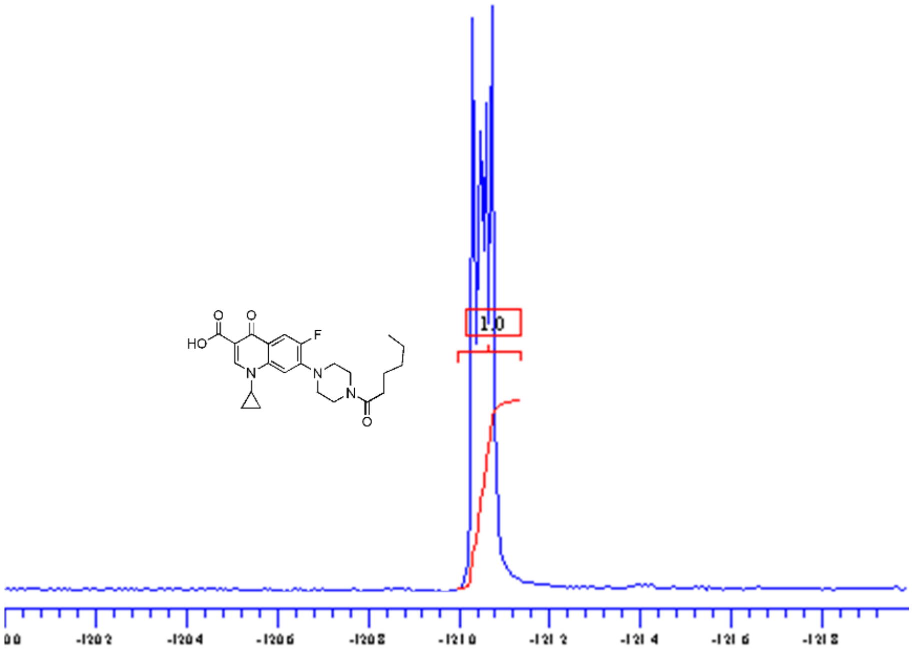

Figure S2. ^{19}F-NMR spectrum of CPF-hex (1-cyclopropyl-6-fluoro-7-(4-hexanoylpiperazin-1-yl)-4-oxo-1,4-dihydroquinoline-3-carboxylic acid).

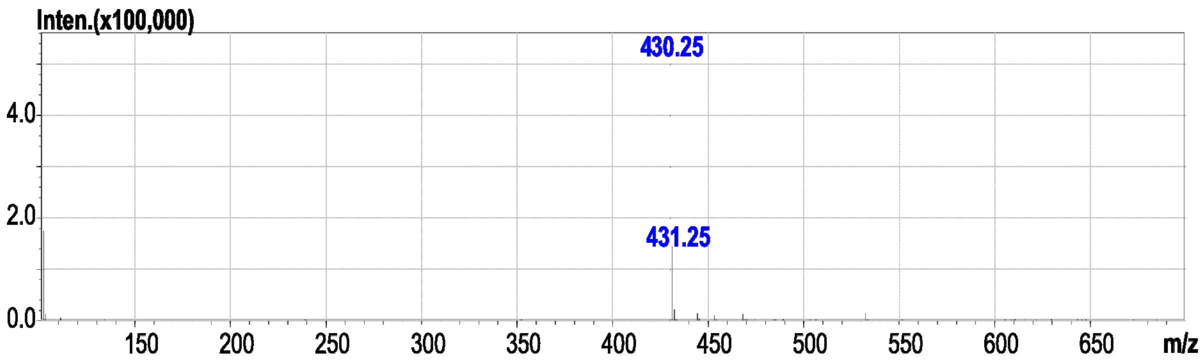

Figure S3. ESI-MS spectrum of CPF-hex (positive ions registration mode).

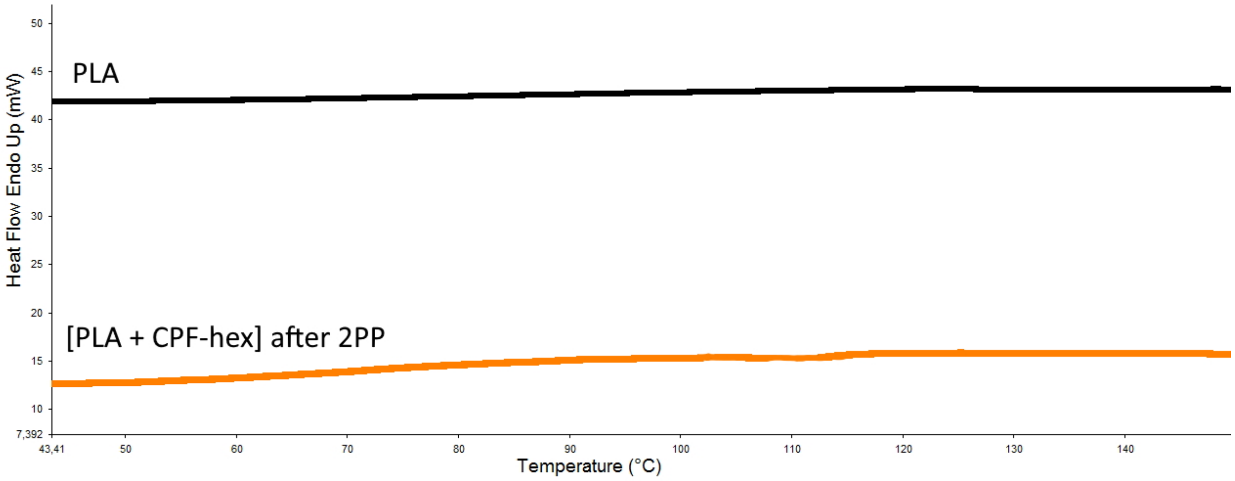

Figure S4. DSC curves of initial methacrylate-terminated star-shaped polylactide (PLA) and a polylactide-based resin after the 2PP processing ([PLA+ CPF-hex] after 2PP) (the second scan).
